# Mechanical Disabilities of the Feet

**Published:** 1919-10-18

**Authors:** 


### MECHANICAL DISABILITIES OF THE FEET.

### The First of Two Post-Graduate Lectures.

The above formed the subject of the first of a series of lectures being delivered at the Royal Society of Medicine in connection with the newly-established Fellowship of Medicine. The lecturer was Mr. W. H. Trethowan, of Guy's Hospital and the Military Hospital, Shepherd's Bush, and he demonstrated patients, and illustrated his thesis by a series of plaster casts. The lecture itself was divided under the following headings: (1) Weak or flat feet; (2) conditions in which the ankle or the arch gives way; (3) the rigid foot with too much arch; (4) abnormalities of the anterior arch. We make the following summary of his main points.

In dealing with foot trouble it is of the first importance to ascertain clearly the kind and degree of disability. Deformity is secondary in importance to disability. All orthopædic surgery turns on the condition of the joints; restriction of movement is a clear indication for treatment. If a joint is stiff and the nerves are intact, muscular power is of secondary importance; the latter may be weakened owing to disuse atrophy. If the joint is movable, muscular power must return. Should movement not be restricted and there be no nerve discontinuity, pain may be ignored. The movement of the ankle joint is purely one of dorsiflexion and plantiflexion, and these are not in a true plane with the leg: the real direction is upwards and outwards to downwards and inwards. The ankle joint should have an 80° range of movement.

With regard to muscular action, the foot has two separate functions: it is a muscular organ whereby its possessor transfers himself from place to place, it also serves as a pedestal for standing firmly when stationary. The structure of the foot alters according to which of these uses it is put to.

The chief leverage action of the foot is that of plantiflexion in the act of walking, and is performed largely by the gastrocnemius and other calf muscles. In raising the foot in walking the longitudinal arch is increased.

A foot must be examined in regard to its power of dorsiflexion; this must be done when the foot is a little inverted. The big-toe joint must be specially attended to in the examination. When the foot is being rested upon, the muscles are relaxed, and the tendons or ligaments come into play. When the inner arch of the foot is straightened the difference takes place at the sub-astragaloid joint; the front of the astragulus and the front of the os calcis tend to turn inwards and a little downwards. Hence the deltoid and interosseous ligaments become tightened, leaving the big muscles more or less at rest. The flattening of the foot is really due to a gliding of the joints on one another until they lock, and are so held by tight ligaments. A man standing in a resting posture places his legs apart, feet somewhat turned outwards, and the trunk thrown backwards. When ready for action, as in starting to run, the feet are closer together, toes turned inwards, and the trunk thrown forwards, causing the leg and buttock muscles to be taut.

It is very important that rest and activity should alternate with one another. People whose leg and foot muscles are over-tired, cease to use them fully in walking, and the feet are used as props. Ligaments used unduly stretch and eventually give way.

The essence of treatment for foot disabilities is to secure proper postural alternation—activity and rest. By using the foot when it is locked, the small joints are irritated, synovitis ensues, and osteophytes form as the result of the joint edges being impinged upon. Alteration of posture is not a necessity of progression, but it is a need for good health.

The patient must be instructed to use the feet muscularly, and not merely as stumps. All persons with weak feet persist in the adoption of the passive attitude, and this means that the ligaments do not receive their due period of rest, but usurp the function of the muscles, and there is a dropping of the longitudinal arch.

Flat-foot is not, of itself, disabling; disability arises from the valgus deformity and the dropping of the arch. Normally, every person is, in some degree, knock-kneed and knock-ankled: the weight of the body is not communicated in a straight line.

It is the muscles which, in strain, give out first. Later, if correction has not been established, the strain is borne by the ligaments, and there is acute pain on the inner side of the foot, with stretching of the plantar fascia and restricted movement. The stiffening brings on cold feet and sweating.

The first aim of treatment is to relieve the stiffness. An acutely stiff foot needs rest; such a patient should be taken right off his feet for a time. In an early stage a week or two of rest and massage may clear up the disability, as it may have been merely muscular spasm. If the condition has persisted for some months, synovitis, adhesions, and joint changes will have been set up, followed by arthritis; there will occur eversion, and the inner curve of the foot will be over-stretched and weak.

Mere flat-foot without pain does not necessarily demand treatment. A man with painless flat-foot has probably already passed through the painful stage, and an elaborate operation for such a case does not seem justified. A very painful foot may not show much structural change; only the muscles are beginning to tire and the ligaments to stretch.

Manipulation of a foot under general anæsthesia will enable a judgment to be formed as to the possible range of movement, and then a decision as to what is best can be arrived at. If movement is found to be definitely restricted under gas, resort must be had to tenotomies.

In very pronounced flat-foot, adduction is first corrected: attention is paid first to inversion, then to dorsiflexion, afterwards using a splint. The essence of the treatment is re-education; the patient must have restored to him good habits of walking.

**Mechanical Disabilities of the Feet**—(*continued*).

Mechanical support may be needed for a time. Deformity may be counteracted by wearing a boot having the inside of the sole definitely thicker than the outside so as to throw the foot continually outward, to counteract the deformity. But every effort should be made to get the foot soft: a stiff foot will largely cancel all these efforts. If the bones are moving well exercises will bring back the muscular power. The patient must be taught to walk with the outer sides of the feet, keeping the feet straight, not at an angle. The action of each foot will then end on all the toes, instead of almost entirely on the great toe. Exercises for a few minutes daily are useless if a proper way of walking is not acquired.

Some hold that heels of boots should always be low, but this the lecturer does not agree with; he considers that for men heels should be definitely higher than is generally the case, certainly more than an inch. The chief reason is that dorsiflexion and plantiflexion are not straight up-and-down movements, but down-and-in to up-and-out. Mr. Trethowan gave some interesting points about walking. Nothing helps to ensure a proper use of the musculature of the feet and a springy step so much as short steps, with the feet straight out, not held at an angle. By taking long strides the pedestrian can easily swing the leg as a whole from the hip, and use the feet more or less as mere stumps. He regards the length of the stride as a function of the fitness of the foot.

The prevailing fault in boots is that they are too narrow at their broadest part; usually, too, the ends are too pointed, resulting in an undue compression of the toes. Proper flexion of toes will do much to restore the integrity of the foot arch.

With regard to hallux valgus and hallux rigidus, it is essential that the patient shall be able to dorsiflex the great toe. It may not be necessary to give him a new joint if a portion of the joint remains. Often dorsiflexion is much improved by removing a small wedge from the metatarsal end of the bone.

